# Age similarities in the anchoring effect in emotion intensity judgment

**DOI:** 10.1186/s40359-023-01101-w

**Published:** 2023-05-15

**Authors:** Menghan Jin, Huamao Peng, Dahua Wang

**Affiliations:** 1 Institute of Developmental Psychology, Beijing Normal University, Beijing, China; 2 Beijing Key Laboratory of Applied Experimental Psychology, Beijing Normal University, Beijing, China

**Keywords:** Age Similarity, Anchoring Effect, Emotional Judgment, Judgment Bias

## Abstract

**Background:**

The anchoring effect refers to the tendency that an individual’s numerical judgment would assimilate to an anchor (a numerical value) that appears before that judgment. This study investigated whether the anchoring effect exists in the emotion judgment of younger and older adults and observed the age-related characteristics. This could not only broaden the explanation of the anchoring effect but also link this classic judgment bias with daily emotion judgment to refresh our understanding of older adults’ ability in emotional perspective taking.

**Method:**

Participants (older adults: n = 64, age range: 60–74, 27 males; younger adults: n = 68, age range: 18–34, 34 males) read a brief emotional story and compared the protagonist’s emotion intensity to a given numerical anchor (lower or higher than the anchor) and then estimated the protagonist’s possible emotion intensity in that story. The task was divided into two cases according to anchor relevance (anchors are relevant or irrelevant relative to the judgment target).

**Results:**

The results showed that the estimates were higher under high-anchor than low-anchor conditions, suggesting the robust anchoring effect. Further, the anchoring effect was greater for anchor-relevant than anchor-irrelevant tasks and for negative rather than positive emotions. No age differences were found.

**Discussion and conclusions:**

The results indicated that the anchoring effect is robust and stable for younger and older adults, even though the anchor information seemed irrelevant. Finally, perceiving others’ negative emotions is a crucial but rather difficult aspect of empathy, which could be a challenge and requires more caution for accurate interpretation.

**Supplementary Information:**

The online version contains supplementary material available at 10.1186/s40359-023-01101-w.

## Introduction

People’s judgments of others’ emotions are sometimes biased, which may cause interpersonal problems, such as misunderstandings among friends [1]. Several studies have indicated that contextual information could affect the accuracy of an individual’s emotion recognition [2–5]. However, much of the research about emotion judgment has focused on emotion category judgment, with relatively rare explorations of emotion intensity judgment, which is more challenging than category judgment [6].

The present study sought to explore emotion intensity judgment bias and related contextual factors, which refer to anchors in the present study. Additionally, age differences in the emotion intensity judgment bias were considered, as cognitive resources decrease and emotional motivation changes with age [7–9], which may influence older adults’ emotion judgment.

Emotion perception, from facial expression perception to socio-emotional processing, is described as a type of predictive processing that emphasizes the match between the prediction generated by the brain in a certain context and the actual sensory input [10]. In this process, contextual information and prior knowledge are presumed to influence the emotion perception result. Research has shown that perceivers’ judgments of facial emotions would be affected by several contextual factors, such as the description of the social situation [11], voices, body postures, and visual scenes [8, 12, 13]. Context stimuli are not the only factors influencing emotion perception. Perceptual knowledge about emotion (the knowledge and understanding of the psychological meanings of different emotions) is also essential for emotion perception [3]. Thus, emotion intensity judgment is likely to be influenced by contextual information and perceivers’ prior knowledge. For instance, a perceiver may misjudge a person’s sadness intensity if the perceiver just watched a sad movie. This judgment bias could be linked to the anchoring effect that highlights how contextual information (i.e., anchors) biases people’s judgment.

Anchoring effects occur when judgment is affected by a specific value that appears before the judgment and causes the estimates to assimilate to the anchor [14]. For example, participants’ estimations about the protagonist’s positive emotion level in a story varied with the high or low estimation value given in an answer to a presented sample [15]. The existence of the anchoring effect has been repeatedly confirmed in various contexts, not only in judgments with numerical answers—such as risk and uncertainty assessments [16], purchase intentions [17], judgments of confidence [18], and work performance assessments [19]—but also in some social judgments with no objective or absolutely correct answer, such as the judicial judgments of experienced legal experts [20], individual estimates of the occurrence of probabilities of social life events [21], and evaluations of facial attractiveness [22]. These social judgments about other people are considered to be similar to emotion judgment, especially in the study about the anchoring effect in perspective taking [23]. The connection between the anchoring effect and emotion judgment is a relatively new research field and lacks empirical study. However, recently, one study found that perceivers’ emotional judgment regarding the degree of positive emotions of a protagonist was affected by the positive/negative additional contextual information they received about the scenario [24]. Another study conducted in China explained the interpersonal empathy gap between teachers and students using the mechanism of the anchoring effect [25].

Apart from limited studies on the anchoring effect of emotion judgment, to date, no study has directly analyzed the effect of age on the anchoring effect of emotion judgment. Older adults seem to be at a disadvantage in terms of the anchoring effect, considering the characteristics of cognitive aging. The insufficient adjustment model states that the anchor is regarded as the starting point of judgment and it then adjusts to a seemingly plausible estimate [14]. Insufficient adjustment by the anchor could be caused by the lack of task motivation or cognitive resources [14]. Thus, the deterioration of cognitive resources among older adults, such as reduced processing speed and decreased memory [8, 9, 26, 27], might place them in a more susceptible position in terms of the anchoring effect.

Most of the early research that indicated the poorer performance of older adults in emotion recognition, used traditional emotion valence or category identification tasks [28–31]. These tasks were not related to the social context of personal life and relied primarily on the ability to detect face feature details. Emotion judgment based on textual description has been one of the most common platforms in emotion research [32–34]. In daily life, more and more interpersonal interactions and corresponding emotion judgments occur through textual information (e.g., email and social media). Moreover, the model of strength and vulnerability integration states that the time older adults have lived and the time they have left would help them gain more emotion regulation strategies, which also include those related to better emotion judgment skills [35]. In addition, several empathy abilities are well-maintained till the age of 60–69 and are comparable with those of younger adults [36]. Empathy refers to the ability to understand others’ thoughts and feelings, containing both cognitive and emotional components [37]. Judging others’ emotions under the interference of an emotional anchor not only requires cognitive empathy ability (the ability to understand and imagine others’ situations and inner states) [34, 38] but also relies on emotional empathy ability (the ability to be influenced by others’ emotions, such as the emotional anchor in the present study) [37]. Hence, we inferred that the well-maintained empathy abilities of older adults may balance the disadvantage of their cognitive decline in the anchoring effect related to emotion judgment about others. Further, past research has found that older adults perform worse in negative emotion perception and judgment compared to younger adults [28, 38–41]. A study on the hindsight effect also found that older adults’ hindsight bias (that the recall judgment of an original response on certain questions would be biased because of the presented correct answer) was more pronounced for negative outcome tasks compared to positive outcome tasks [42]. Based on these findings, this study infers that age similarities in the anchoring effect may be found in the emotion judgment about others, and if there are age differences in the anchoring effect, they are more likely exist in negative emotion judgment.

To better understand and interpret an individual’s performance and the age characteristics related to emotion judgment under a biased emotional anchor, this study measured some background factors to test their relationship with the individual’s anchoring effect tendency. The abilities that reflect limited cognitive resources (such as processing speed and working memory [8, 9]) were taken into account. Moreover, empathy was measured because of its close relationship with the emotion judgment task.

In sum, this study aimed to investigate whether there are age differences or age similarities in the anchoring effect of emotion intensity judgment by adopting life-like scenarios that captured interpersonal emotions combined in the classic anchoring effect paradigm. Besides the impact of the perceiver (age-related influence) on the anchoring effect, the impact of the emotional anchor information itself should be considered. Anchor relevance was explored in the present study; the relevance between the anchor information and the judgment target was manipulated. Based on the selective accessibility model [15], relevant anchoring information could provide directly available referential cues for judgment. For instance, when the participants were informed of the causal relationship between the contextual information and the target emotion, they demonstrated significantly higher accuracy in their judgment of the target emotion than in all irrelevant contexts (no such relationship existed) [39]. However, there is not much evidence about whether an irrelevant anchor can bias emotion judgment. If irrelevant anchor information could still bias participants’ judgments, this probably indicates that the anchoring effect in emotion judgment is non-negligible in daily interpersonal life. Thus, the present study further investigated the impact of anchor relevance on the anchoring effect in emotion judgment.

### Overview of the present research

The present study aimed to investigate whether emotional anchor information affects emotion judgment, while also exploring possible age-related differences or similarities.

The classical comparison-judgment two-step paradigm of the anchoring effect was employed in the present study [14]. Specifically, the present study investigated the anchoring effect in emotion judgment while focusing on the impact of anchor relevance, which was manipulated using consistent/different interpersonal scenarios and protagonists for the anchor and target. For example, the anchor was highly relevant to the judgment target if the anchor and target scenarios were all related to the same protagonist’s emotion in a consistent emotional event (e.g., all about someone’s anger emotion intensity after being deceived by others, which is an example used in the experimental task). Further, the anchor was nearly irrelevant to the judgment target if the questions for the anchor and the target inquired about the emotions of different protagonists in two different scenarios (e.g., anchor: protagonist A’s certain emotion intensity after he encountered an emotional situation A; judgment target: protagonist B’s certain emotion intensity after he encountered an emotional situation B, this is an example schema used in the experimental task).

### Hypotheses for the present study

Based on the discussions above, this study proposed the following hypotheses:

#### Hypothesis 1

An anchor affects participants’ emotional judgment: Participants’ estimates are significantly higher under the high-anchor condition than under the low-anchor condition.

#### Hypothesis 2

Anchor relevance affects participants’ anchoring effect: Participants are more affected by an anchor under the anchor-relevant condition, relative to the anchor-irrelevant condition.

#### Hypothesis 3

Age differences or age similarities in the anchoring effect in emotion judgment exist. Age differences are more likely to exist when judging negative emotions; older adults’ estimates are more affected by the anchor and the characteristic of the anchor (anchor relevance) than those of younger adults when judging negative emotions.

## Method

### Participants

Sixty-four older adults (age: *M* = 64.62, *SD* = 3.82; education year: *M* = 10.89, *SD* = 2.67; 27 males) and sixty-eight younger adults (age: *M* = 22.21, *SD* = 2.89; education year: *M* = 16.17, *SD* = 1.79; 34 males) were recruited. The sample size was decided using G*power 3.1 [43]. Specifically, the F-test for analysis of covariance (ANCOVA) was selected, with an effect size f^2^ of 0.57 [24], an alpha of 0.05, a power of 0.95, four groups, and 2 covariates (see analysis plan). The results indicated that a total sample size of 85 individuals was required. Approximately 30 participants were recruited for each condition considering the possible depression screening. Older adults were recruited from communities in Beijing, and younger adults were recruited from Beijing Normal University and some nearby universities. This study only recruited participants with healthy physical and psychological states (no serious affective problems and cognitive impairment, such as depression symptoms and extremely low cognitive performance), who were screened using depression scales and cognitive tests.

The descriptive results of the background variables of younger and older adults are shown in Table 1. Sixty-two older adults (excluded two participants) and sixty-seven younger adults (excluded one participant) were retained as valid participants after screening for depression.

**Table 1:** Description of the demographic variables of younger and older adults

	High-anchor group (n = 64)				Low-anchor group (n = 66)				Effect of anchor			Effect of age		
	Old (n = 31)		Young (n = 33)		Old (n = 31)		Young (n = 34)		*F (df)*	*p*	η_p_^2^	*F (df)*	*p*	η_p_^2^
	*M*	*SD*	*M*	*SD*	*M*	*SD*	*M*	*SD*						
Age	64.60	3.78	22.08	3.20	64.80	4.13	22.48	2.34	0.34(1,120)	0.564	0.003	4930.23(1,120)	0.000***	0.976
Education	11.40	2.69	15.92	1.87	10.12	2.49	16.59	1.30	0.04(1,120)	0.836	0	168.62(1,120)	0.000***	0.584
Health	3.73	0.52	3.56	0.58	3.84	0.69	4.07	0.59	7.46(1,120)	0.007**	0.059	0.14(1,120)	0.712	0.001
Processing speed	24.32	5.91	43.09	7.12	21.27	7.10	41.06	6.03	4.19(1,120)	0.043*	0.034	258.76(1,120)	0.000***	0.683
Working memory	5.23	1.63	7.13	1.70	4.27	1.20	7.29	1.72	2.26(1,120)	0.135	0.018	71.95(1,120)	0.000***	0.375
Empathy	57.42	8.30	57.19	8.64	56.67	8.75	56.91	9.39	0.02(1,120)	0.881	0	0.10(1,120)	0.751	0.001

*Note.* The ANOVA results are shown in the columns on the right: “Effect of anchor” represents the related statistic from the main effect of the anchor group, and “Effect of age” represents the related statistic from the main effect of the age group. The significance of the main effect of the anchor group and age group: *, **, and *** indicate *p* < .05, *p* < .01, and *p* < .001, respectively. Self-rated health condition (Health) is rated on a five-point scale (1 = very poor; 2 = poor; 3 = average; 4 = good; and 5 = very good)

A 2 (age: young, old) × 2 (anchor: high, low) two-way ANOVA on the background variables was performed to test the difference between different groups. The results of the main effects of the anchor group and the age group are presented in Table 1. These results indicated that not only were older adults significantly older than younger adults, but they also had fewer years of education and lower performance regarding processing speed and working memory. Moreover, the two anchor groups only showed significant differences in self-reported health and processing speed. Even though there were some significant group differences for some variables, cognitive might be linked to the age effect in the anchoring effect as we have discussed in the introduction. Possible age-related effects can be covered in later analyses if they are controlled as covariates. In addition, no significant correlation was found between cognitive ability variables and judgment scores (both the original estimate and the accuracy index) in the experimental tasks. The correlations with the original estimate were as follows: *r*_speed_=0.02 (*p* = .819), and *r*_memory_=-0.04 (*p* = .640). The correlations with the accuracy index were as follows: *r*_speed_=-0.05 (*p* = .593), and *r*_memory_=-0.04 (*p* = .672). Therefore, processing speed and working memory were not included as covariates in later analyses. Years of education and self-reported health were controlled as covariates in further analyses.

### Measures

The background variable measures mainly included demographic variables: age, gender, years of education, self-rated health condition, and family income (per month).

#### Emotion Judgment Task

**Fig. 1:**
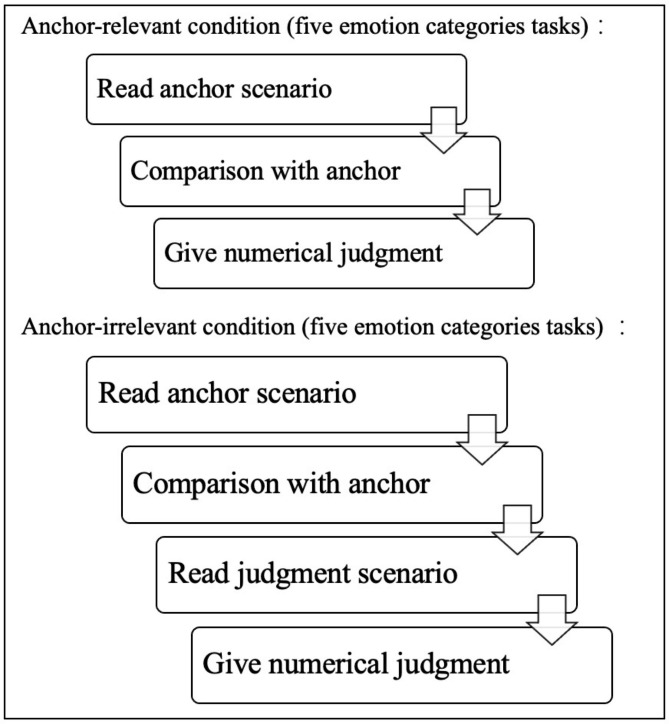
Process of the interpersonal emotion judgment task of the experiment Note: The flow chart illustrates the process of the task under the specific conditions of the experiment. All participants should complete the anchor-relevant condition and anchor-irrelevant condition, and each of these conditions contains tasks of five emotion categories. Each emotion category contains two trials

Figure 1 illustrates the procedure of the emotion judgment task. All tasks started with reading an emotional anchor scenario in the comparison phase in which the participants compared the protagonist’s emotion intensity with a given numerical anchor. Next, in the judgment phase, they provided a judgment estimate of the protagonist’s emotion intensity on a 0 (not at all)–100 (extremely) scale. Samples of the task material in the two conditions are shown in Table 2. In *the anchor-relevant condition*, all comparison and judgment phases shared the same scenario. In *the anchor-irrelevant condition*, the judgment phase was based on a new scenario and a new protagonist but described the same emotion category; hence, the anchors were less useful for subsequent estimates. Two blocks of experimental tasks (anchor-relevant and anchor-irrelevant blocks) were designed. Each block contained 10 trials (two trials for each emotion to balance the protagonist’s gender), and the entire formal task contained 20 trials.

**Table 2:** Sample of materials used for the anchor-relevant and anchor-irrelevant conditions

	Anchor-relevant material sample	Anchor-irrelevant material sample
Scenario of the comparison phase	Mr. Zhang turns 70 years old this year. He is concerned about healthcare products because of his poor health. He just realized that the health product that Mr. Wang persuaded him to buy last week was fake.	Mr. Zhu has just retired. He rides his bike to the food market every day. One day, after he finished shopping, he found that his bicycle, which he had parked on the side of the road, had been knocked down by someone.
Comparison phase	Do you think the anger emotion intensity felt by the protagonist in the scenario is higher or lower than 92?	Do you think the anger emotion intensity felt by the protagonist in the scenario is higher or lower than 87?
Scenario of the judgment phase	(Identical to the comparison phase)	Ms. Zhou has been retired for several years, and she is always concerned about financial products. However, she lost nearly half of her principal last week because of a fraudulent product introduced by someone.
Judgment phase	In your opinion, what is the anger intensity felt by the protagonist in the scenario?	In your opinion, what is the anger intensity felt by the protagonist in the scenario?

*Note*. The scenarios are examples of high anchor and older adult tasks, with joy being the target emotion

The anchors of emotion intensity in the comparison phase were decided based on the rating results of a calibration group, which provides a rating without any anchor. The calibration group included sixty-one older adults (age: *M* = 65.08; *SD* = 6.48; education year: *M* = 10.41, *SD* = 2.85; 23 males) and sixty-three younger adults (age: *M* = 22.02; *SD* = 3.28; education year: *M* = 14.87, *SD* = 2.64; 30 males). The 5th percentiles of the distribution of estimates in the calibration group were selected as the low anchor means, and the 95th percentiles were selected as the high-anchor means in each group. The mean emotion intensity rating of target scenarios from the calibration group and the high and low anchor for the two age groups and for positive emotion and negative emotion are presented in Table 3.

**Table 3:** Mean high and low anchor for two younger and older adults

		Mean target emotion intensity rating *M(SD)*	Mean high anchor *M(SD)*	Mean low anchor *M(SD)*
Older adults	Positive emotion	67.88(11.07)	86.25(3.01)	46.63(3.85)
	Negative emotion	66.40(14.16)	90.17(2.98)	40.75(3.02)
Younger adults	Positive emotion	67.78(11.32)	86.25(4.06)	45.50(3.66)
	Negative emotion	65.84(15.04)	88.42(4.06)	36.28(3.70)

The emotion categories and emotional scenarios for younger and older adults were selected and compiled based on interviews in the pilot study with a group of younger and older adults that concerned emotional events in their daily life (e.g., “children returning home and visiting parents” for older adults and “making new friends” for younger adults). According to the frequency of each emotion category being mentioned in the interviews, joy, and pride were selected to represent positive emotions, and anger, distress, and sadness were selected to represent negative emotions, for which corresponding scenarios were also selected. The calibration group in the pilot study rated the emotion intensity felt by the protagonist in the scenario, and also rated the familiarity and importance of the scenario (on a seven-point Likert scale; from 1 = Not familiar/important at all to 7 = Extremely familiar/important) to ensure the consistency of experimental scenario materials for the two age groups. Finally, a set of materials was screened and selected for the formal experiment, ensuring the materials for older and younger adults are comparable and are similar in several reported scenario characteristics for each age group. Specifically, the calibration group’s ratings of the scenario materials had nonsignificant differences in age regarding the rating of intensity, familiarity, and importance. The detailed information about the pitot study and the descriptive information on the ratings of younger and older adults in the final screened positive and negative scenarios are presented in Supplement [see Additional file 1].

#### Depression

The present study also tested depression among older and younger adults, to exclude any participants with serious and salient depression symptoms, which might noticeably affect their emotional perception and judgment results. Depression among older adults was measured using the Geriatric Depression Scale (GDS-15; [44]). This scale measures the feelings of older adults during the past week, with 15 items and a rating of 1 (yes)/0 (no). The Cronbach’s α coefficient of the scale is 0.82. The variance factor of depressive experience and positive emotion was 43.21% [45]. The Cronbach’s α coefficient of GDS-15 in the older adults sample of the present study was 0.63, which is acceptable considering the relatively small sample (62 older adults). Depression among younger adults was measured using the Center for Epidemiologic Studies Depression Scale-13 [CES-D-13; 46]. The scale measures the frequency of certain symptoms over the previous week and contains 13 items scored on a four-point rating scale (0 = less than 1 day; 1 = 1–2 days; 2 = 3–4 days; 3 = 5–7 days). Higher scores indicate a higher degree of depression. The Cronbach’s α coefficient of the scale is 0.87, and the scores of CES-D-13 were significantly correlated with emotional experience (r=-.69, p < .01), and sleep quality (r = .41, p < .01) [46]. The Cronbach’s α coefficient of CES-D-13 in the younger adults sample of the present study was 0.67, which is acceptable considering the relatively small sample (67 younger adults). We used the outlier standard (the score must be more than 2.5 standard deviations above the intragroup mean score) as a screening standard for older and younger adults.

#### Empathy

The present study used the Interpersonal Reactivity Index (IRI-C) to measure empathy levels [37]. This scale contains four main factors: Perspective taking (the tendency of adopting others’ ideas [37]), Fantasy (the ability to imagine and sympathize with fictional characters [37]), Empathic Concern (the ability to be concerned about and sympathize with others’ affects and emotions, [37]), and Personal Distress (the tendency of feeling anxious and distressed in a tense interpersonal situation [37]). The scale includes 22 items rated on a five-point Likert scale (0 = inappropriate to 4 = very appropriate). Subscale scores are summed item scores, and a higher score indicates a higher dimensional level. Scale scores are summed subscale scores, and a higher score indicates a higher empathy level. The retest reliability of the scale was 0.737, and the retest reliability of the subfactors (perspective taking, fantasy, empathic concern, and personal distress) were 0.700, 0.735, 0.625, and 0.655, respectively. These four factors explain 46.342% of the total square deviation and exhibit good cross-sample consistency and differentiation (p < .001) [47]. The Cronbach’s α coefficient of the IRI-C in the sample of the present study was 0.82.

#### Processing speed

Processing speed was measured using the letter comparison task. The participants judged whether two pairs of digit strings were identical (e.g., 482–482 and 658,331–656,331) in the allotted time (90 s). The split-half reliability of the letter comparison task was r = .94 [48]. The score was measured by the maximum number of items completed correctly during the allotted time. A higher score indicated a higher information processing speed. Forty-eight trials were performed in the present study. The range of potential scores was 0–48.

#### Working memory

Working memory was measured using the backward digit span task from the Wechsler Adult Intelligence Scale, 3rd edition [49]. The split-half reliability of the backward digit span task was r = .89[48]. A series of digit strings were read to the participants, who were asked to recite the strings backward. Working memory span was measured by the maximum number of digit strings one could repeat correctly. The higher the number of the digit strings one could repeat correctly, the better one’s working memory ability. Ten trials were performed in the present study. The range of potential scores was 0–10.

The screening standard for cognitive abilities was that the participants with both a processing speed score and a working memory score that were outliers (the score must be lower than 2.5 standard deviations above the intragroup mean score) would be screened out. No older adult or younger adult was screened out from the final data according to this criterion.

### Procedure

The present study was conducted in accordance with the recommendations of the Ethics Committee of the School of Psychology, Beijing Normal University, and approved by the same committee. All the participants gave written informed consent in accordance with the Declaration of Helsinki.

After providing informed consent, the younger and older participants first completed the demographic information questionnaire. Next, they were randomly assigned to either the high-anchor or low-anchor group, with half of the participants in each group receiving materials in Sequence 1 and the other half receiving materials in Sequence 2. Sequence 1 started with the anchor-relevant condition followed by the anchor-irrelevant condition, and Sequence 2 followed the reverse order. Approximately 30 participants were recruited under each condition in the formal experiment.

To ensure that the high-anchor and low-anchor groups were not systematically different in terms of judging tendency, the participants were required to complete a baseline emotion intensity judgment task before the formal experimental task. The baseline task contained five trials (one trial for each emotion). The results from the baseline check verified that there was no significant difference in judgment tendency between the two anchor groups: *M*_*high*_=70.25 ± 13.70; *M*_*low*_=70.89 ± 14.20, *t* (131) = 0.264, *p* = .792, *MD* = 0.64, Cohen’s d = 0.05. Therefore, the confounding effect of judgment tendency could be excluded. One practice task was completed before the formal task to ensure the participants understand the task.

### Study design

The present study contained two age groups. In each age group, the participants were randomly assigned to one of two anchor groups (high or low anchor group). Anchor relevance referred to the relevance between scenarios in the comparison and judgment phases. All the participants had to complete emotion judgment tasks under two conditions: anchor-relevant and anchor-irrelevant. In addition, the emotion in the tasks contained two emotional valences. Emotional valence represented the valence (positive or negative) of the emotion felt by the protagonist. Positive emotions included joy and pride, and negative emotions included anger, distress, and sadness, based on pilot interviews with older and younger adults.

In the experiment, all the participants had to read an emotional scenario and compare the protagonist’s emotion intensity with the anchor, and then give a numerical estimate of the protagonist’s emotion intensity. The direct dependent variable was the participant’s final numerical estimate (scale of 0 [not at all] to 100 [extremely]) for the protagonist’s emotion intensity in the reading scenarios. The anchoring effect was the significant difference in the mean estimates between the high and low anchor conditions. Higher differences indicated a greater anchoring effect. The judgment accuracy index was generated as the absolute difference between the participant’s estimate of the target scenario and the mean estimate of the calibration group for the same scenario. This was calculated using the following formula: the judgment accuracy index= | the numerical estimate for the target scenario – the mean rating of the calibration group for that scenario |. For example, if one participant’s estimate for one of the target scenarios was 90, and the mean estimate of the calibration group for this scenario was 70, then the judgment accuracy index for this participant was 20. The calibration group rated scenarios in a normal context without any anchor condition, which resulted in relatively unbiased ratings. Detailed information about the calibration group is presented in Supplement [see Additional file 1].

### Analysis plan

Two analysis indexes were used to investigate the impact of the emotional anchor on judging others’ emotions. These indexes reflected whether and how an individual would be affected by the emotional anchor when judging others’ emotions. The first index was the emotion intensity estimate for the judgment target. The estimates for each item of each participant were collected. The anchoring effect was indicated by the significance of estimate differences between the high and low anchor conditions. This index focused on whether the anchors affect one’s judgment. The second was the judgment accuracy index. This index was calculated by generating the absolute difference between the participants’ numerical estimates and the mean rating of the calibration group for each target scenario. Specifically, a higher absolute difference meant lower accuracy of emotion judgment. The second index measured the extent to which a high or low anchor impacts judgment.

The accuracy index was assessed at an individual level; therefore, it could better investigate the relationship between individuals’ tendencies of biased judgment and background variables (cognitive abilities and empathy). Thus, the correlation between the accuracy index and background variables was analyzed to further interpret the factor influencing individuals’ tendencies of biased judgment and its relationship with age. Finally, the data were also analyzed by ANCOVA using SPSS ver. 26, with repeated measures to detect the main effects and interactions on the mean estimates and accuracy indexes. A 2 (age: young, old; between-subject) × 2 (anchor: high, low; between-subject) × 2 (emotional valence: positive, negative; within-subject) × 2 (anchor relevance: anchor-relevant, task-irrelevant; within-subject) repeated-measures analysis of covariance (ANCOVA) on mean estimates of emotion intensity and on the judgment accuracy index were performed respectively, with years of education and self-reported health as covariates. The significance level was set at α = 0.05 for all statistical inferences.

## Results

### Anchoring effect analysis

**Fig. 2:**
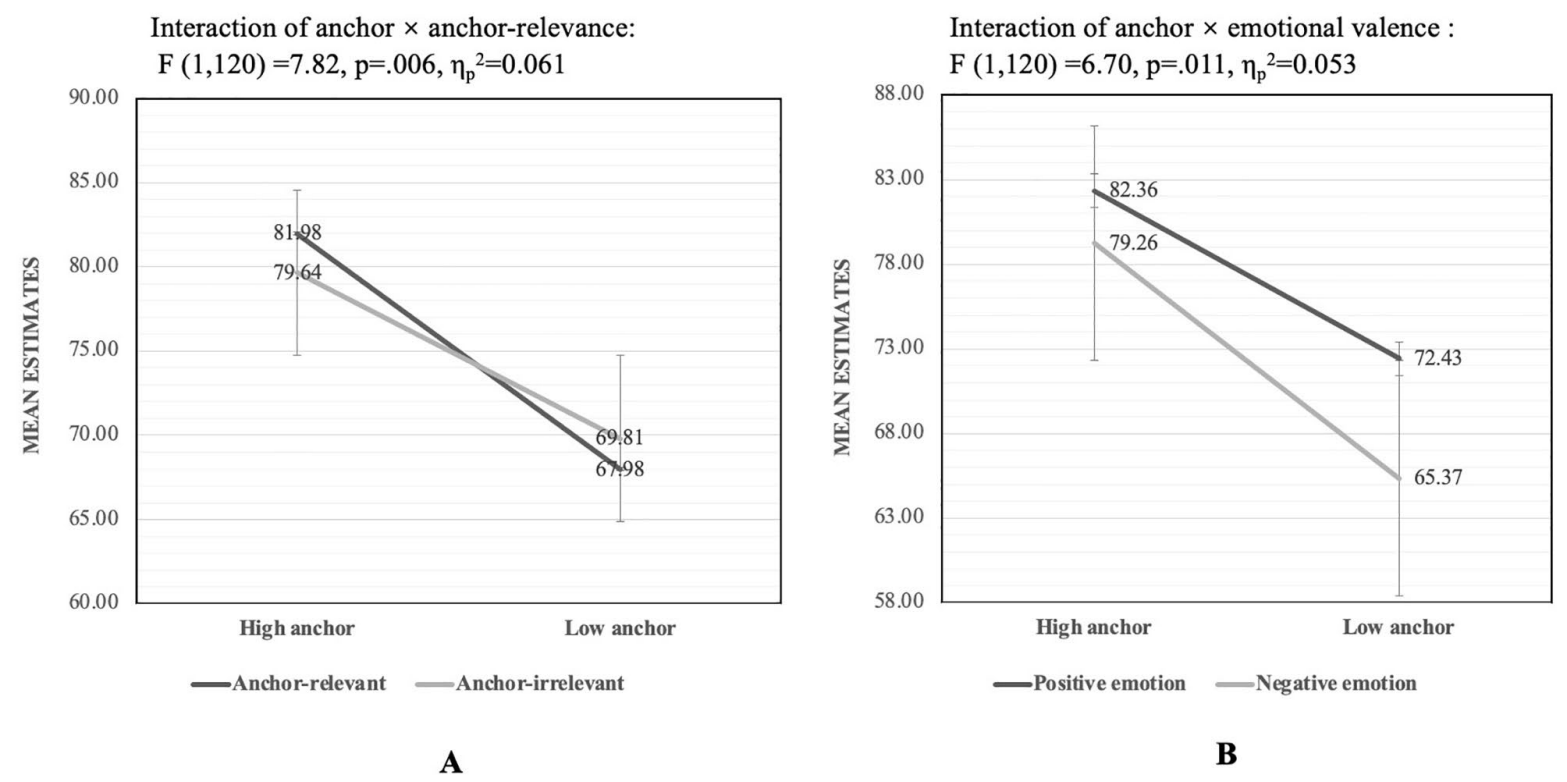
Line chart of the interaction effect on the mean estimates Note: the error bars represent standard errors. Graph 2 A is the interaction between anchor and anchor relevance. And this figure shows that the estimate difference between the two anchor groups is much larger in the condition of anchor-relevant than in the condition of anchor-irrelevant; Graph 2B is the interaction between anchor and emotional valence. And this figure shows that the estimate difference between the two anchor groups is much larger when judging negative emotion than judging positive emotion

The descriptive statistics of the mean emotion intensity estimates of the two age groups are presented in Table 4. The results from ANCOVA indicated a significant anchoring effect, and the estimates under the high-anchor condition were significantly higher than those under the low anchor condition: *F* (1,120) = 39.62, *p* = .000, η_p_^2^ = 0.248, *M*_high_=80.81 ± 10.56, *M*_low_=68.9 ± 11.31. The interaction between the anchor and emotional valence was also significant: *F* (1,120) = 6.70, *p* = .011, η_p_^2^ = 0.053. The simple effects test indicated that the anchoring effects were larger when judging negative emotion (positive: *F* (1,120) = 27.09, *p* = .000, η_p_^2^ = 0.184, *MD* = 10.96; negative: *F* (1,120) = 44.52, *p* = .000, η_p_^2^ = 0.271, *MD* = 14.57). The interaction effect between the anchor and anchor relevance was significant: *F* (1,120) = 7.82, *p* = .006, η_p_^2^ = 0.061. The anchoring effects were larger under the anchor-relevant condition than under the anchor-irrelevant condition (anchor-relevant: *F* (1,120) = 46.31, *p* = .000, η_p_^2^ = 0.278, *MD* = 15.06; anchor-irrelevant: *F* (1,120) = 23.43, *p* = .000, η_p_^2^ = 0.163, *MD* = 10.47). The interaction effects between anchor and anchor relevance, and anchor and emotional valence are illustrated in Fig. 2. The interaction between emotional valence and age was significant: *F* (1,120) = 18.07, *p* = .000, η_p_^2^ = 0.131. The estimates of older adults were marginally higher than those of younger adults in the positive emotion task (*F* (1,120) = 3.27, *p* = .073, η_p_^2^ = 0.027, *MD* = 5.77), but no age differences were found in the negative emotion task (*F* (1,120) = 0.95, *p* = .331, η_p_^2^ = 0.008, *MD*=-3.23). No other significant main or interaction effect was found, including the interaction between age and anchor; three-way interaction between age, anchor, and anchor relevance; and four-way interaction between age, anchor, emotional valence, and anchor relevance. Thus, no age-related differences in the anchoring effect and in the effects of emotional valence and anchor relevance were found.

**Table 4:** Mean emotion intensity estimates of younger and older adults

Anchor relevance	Emotional valence	Age group	Mean emotion intensity estimates		Mean judgment accuracy			
			High anchor *M(SD)*	Low anchor *M(SD)*		High anchor *M(SD)*	Low anchor *M(SD)*	
Relevant	Positive	Older	87.06(8.06)	72.93(17.35)		20.10(6.05)	15.10(11.64)	
		Younger	82.12(12.46)	72.20(10.79)		17.67(6.82)	14.13(5.21)	
	Negative	Older	77.24(12.00)	59.36(15.86)		21.21(6.67)	16.58(9.96)	
		Younger	81.51(12.06)	67.24(12.08)		17.69(5.92)	16.62(6.94)	
Irrelevant	Positive	Older	82.82(11.25)	76.65(11.07)		19.32(6.97)	14.04(6.55)	
		Younger	77.74(12.44)	68.21(15.65)		16.18(6.39)	15.82(8.20)	
	Negative	Older	79.90(11.47)	67.17(9.48)		18.20(5.30)	13.65(5.89)	
		Younger	78.31(14.08)	67.45(14.29)		17.95(7.10)	15.96(8.74)	
Total		Older	81.75(9.06)	69.04(11.65)		19.71(4.94)	14.88(6.39)	
		Younger	79.92(11.87)	68.77(11.16)		17.47(6.00)	15.76(5.80)	

### Judgment accuracy index analysis

The mean judgment accuracy indexes were initially compared to 0, and the one-sample T-test result indicated a significant difference with 0: *t* (128) = 32.46, *p* = .000; thus, the participants’ judgments of targets’ emotions were significantly different from the calibration group’s ratings. The descriptive statistics of the mean judgment accuracy index under different conditions are presented in Table 4. The results from ANCOVA indicated that the main effect of the anchor was significant: *F* (1,120) = 9.28, *p* = .003, η_p_^2^ = 0.072. The accuracy indexes under the high-anchor condition were significantly higher than those under the low-anchor condition, which meant emotion judgment was more affected by a high anchor than a low anchor. The main effect of the age group was not significant: *F* (1,120) = 0.031, *p* = .860, η_p_^2^ = 0, which was consistent with the age similarity result from the anchoring effect analysis. No other significant main or interaction effect was found.

Next, Pearson correlations between the background variables and accuracy index across all experimental conditions were analyzed. The results indicated that the cognitive abilities measured in the present study showed a nonsignificant correlation with accuracy (processing speed: *r*=-.05, *p* = .593; working memory: *r*=-.04, *p* = .672). Only the total score of empathy was positively correlated with accuracy, *r* = .19, *p* = .035, which indicated that the participants with higher empathy exhibited lower judgment accuracy. Further, the correlation between empathy subscale scores and accuracy indicated that the empathic concern score was positively correlated with accuracy: *r* = .22, *p* = .015. However, it was the perspective taking score that positively correlated with age (*r* = .29, *p* = .000), while the fantasy score negatively correlated with age (*r* = -.29, *p* = .000).

## Discussion

The present study investigated the possible anchoring effects on emotion judgment among younger and older adults and studied the effect of anchor relevance. The results showed that the two age groups exhibited similar anchoring effects. Moreover, the anchoring effect was greater for a relevant anchor and when judging negative emotion.

### Presence of the anchoring effect in emotion judgment

The present study confirmed that emotion judgment would be biased by the anchoring effect, which aligns with previous studies about social interpersonal judgment (e.g., perspective taking [23], attractiveness judgment [22], and positive emotion judgment [24]). However, in the present study, the sample included older adults, and various conditions about anchor information characteristics were considered. The results indicated that even while making emotional judgments that rarely have standard and objective answers, individuals are likely to be influenced by contextual clues. The widespread anchoring effect may need to be considered in dept.

### Age-related similarities in the anchoring effect

Although older adults gave higher intensity estimates in the positive emotion task, relative to younger adults, no general age-related differences in the anchoring effect were found. Similar to research supporting age similarities in emotion perception and judgment [28, 41, 50, 51], the present study’s results found age similarities in emotion judgment even with interferential emotional cues. Further, the present study’s result is consistent with the results of a previous study [52] that found age differences in emotion category judgments but age similarities in emotion intensity (degree) judgments. The emotion judgment tasks in the present study depicted textual scenarios that relied less on facial emotion recognition. Considering the decline in older adults’ face feature detection ability [53], textual information might provide them with an increased opportunity to understand the situation and others’ inner emotions, as Phillips et al. found age similarities in their study that used verbal stories tasks [53].

The correlation between the accuracy index and background variables also indicated that cognitive abilities barely correlated with judgment accuracy, although the older groups had lower cognitive performance than the younger groups. However, judgment accuracy was positively related to one’s empathy ability, especially empathic concern, and the reported similar total score of empathy and score of empathic concern between younger and older adults might lead to age similarities in the anchoring effect. The results showed that higher empathic concern led to lower accuracy and a greater anchoring effect in judgment. Empathic concern is representative of emotional empathy [36]. Emotional empathy ability refers to the extent to which a person is influenced by others’ emotions, such as more sensitive emotion contagion and emotional resonance. Thus, we speculate that individuals with higher empathic concern might be more sensitive to exposure to a biased emotional anchor and are more affected by it in subsequent emotion judgment. The correlation results also indicated that the significant relevant empathy abilities were fantasy (older adults showed a lower level of fantasy) and perspective taking (older adults showed a higher level of perspective taking), which are both parts of cognitive empathy [36]. These two cognitive parts did not relate to judgment accuracy. It seems that emotional empathy is more crucial for accurate judgment under the interference of an emotional anchor, but not a cognitive part of empathy. The well-maintained emotional empathy abilities of older adults [36] might protect them from the impact of an emotional anchor.

No interaction between age, anchor, and emotional valence was found. Thus, older adults might not be particularly susceptible to negative emotion as hypothesized. However, considering the relatively limited categories of emotion in both valences, conclusions about the characteristics of older adults’ anchoring effects on negative emotions cannot be drawn.

### Influence of emotional valence on the anchoring effect

Greater anchoring effects were found in judgments of negative emotions than in judgments of positive emotions. For a more in-depth analysis, the variable of emotional valence was explored in terms of emotion category. After replacing emotional valence with an emotion category (five emotion categories presented in the study), the ANOVA results indicated that the emotions of distress and sadness were more susceptible to the anchoring effect. Negative events were found to affect individuals more than positive events [54], and losses were generally experienced more prominently compared to gains [55]. Thus, individuals might be more easily biased when thinking of negative emotions, such as sadness and distress, which relate primarily to losses in daily life. In addition, researchers have stated that individuals have higher attentional involvement when experiencing happiness and pride, and lower attentional involvement when experiencing sadness and boredom (similar to the “distress” emotion in the present study) [56]. Judging others’ emotions also leads to initially speculating about one’s own emotions in a certain event [57]. Hence, lower attentional involvement might bring about a stronger influence of interference information, such as the anchoring effect. These results underscored our need for caution in perceiving others’ distress and sadness. In addition, susceptibility to contextual interference requires greater vigilance in the case of potentially disruptive emotion information, such as the emotional rendering of public events that deviate from facts and the exaggerated displays of emotion in a variety of advertisements.

### Effects of anchor information characteristics on the anchoring effect

The present study verified that anchor relevance affected anchoring bias in emotion judgment, which proves the greater influence of relevant anchor information on emotional judgment. In addition, the results confirmed that even an irrelevant anchor could induce a significant anchoring effect. Many studies have focused more on relevant anchors of judgment. Nevertheless, the anchoring effect from irrelevant, less informative, or unrelated anchor information should not be neglected. A previous study found that both informative and less informative anchors could induce anchoring effects [58]. The influence of seemingly irrelevant information could nonetheless bias judgments. These results remind us to be more cautious about contextual emotional information interference, with regard to both relevant and irrelevant information, to avoid unwanted bias in judging others’ emotions. Further, a high anchor was found to bias participants’ judgment more than a low anchor; this indicates that individuals might be more affected by a higher intensity of emotional contagion, resulting in an overestimation of others’ emotions. This tendency highlights that individuals should be cautious and vigilant amid the high intensity of emotions over broadcasted in various media.

### Implications, limitations, and future research

The results about bias in interpersonal emotion judgment provided a clear picture of the characteristics of the judgment under emotion-provoking situations for older and younger adults and extended the application of the anchoring effect in the interpretation of people’s judgment bias. Moreover, the results provided implications for marketing and sales owing to the possible influence of emotion-provoking stimuli on people’s judgment and decision-making. Finally, these results might help detect individuals with emotion perception problems from these normative patterns in clinical samples. However, several limitations of the present study need further attention and remedy in future research.

First, the emotion categories used in the current study were limited and did not fully capture the characteristics of positive and negative emotions. Future studies should conduct more in-depth research on the impact of emotional valence and emotion categories on emotion perception and judgment. In addition, the emotion category contains both primary emotions (joy, anger, and sadness) and secondary emotions (pride and distress), which might have different perception characteristics across cultures. Thus, one should be cautious about making a cross-cultural generalization about the results.

Second, the descriptive scenario materials used in the present study to evaluate interpersonal emotion judgment could be improved by increasing the ecological validity of the task forms (e.g., video). A nonverbal task form might increase the cognitive involvement of older adults, and the age characteristics in the anchoring effect and interpersonal emotion judgment might be better reflected and explained.

Third, different depression questionnaires were used for screening older participants and younger participants, although most of the participants were considered qualified to complete emotional judgment tasks without serious affective problems.

Finally, the predictive processing framework explained that previous knowledge (top-down modulation) and sensory information (bottom-up stimulation) are both important for emotion processing [10]. Although age similarities in the anchoring effect and judgment accuracy were found, older adults’ prior knowledge and experience were not measured and manipulated in the present study. Thus, besides the emotional anchor information, the role that prior knowledge and experience play in the age characteristic in the anchoring effect in emotional judgment should also be considered in future research (e.g., a self-generated anchoring effect). Exploring the possible role of knowledge and experience (compensating role or interruptive role for older adults) would provide a greater understanding of age characteristics in emotional judgment.

## Conclusion

The present study showed that there is a strong and stable anchoring effect in the process of interpersonal emotion judgment among both younger and older adults. This suggests the likelihood that both age groups would be disturbed and affected by other emotional information and subsequently produce biased judgments when judging the emotions of others. Individuals may be affected most by relevant external information, particularly when judging others’ negative emotions, such as sadness and distress. However, it must be noted that even unrelated external information could bias interpersonal emotion judgments. Both older and younger adults should be cautious about judging other people’s emotions and make judgments based on facts and actual background information.

## Electronic supplementary material

Below is the link to the electronic supplementary material.

Supplementary Material 1

Supplementary Material 2

## Data Availability

The dataset supporting the conclusions of this article are included within the article and its additional file, named dataset.xlsx.
