# Supplementary material for: Age similarities in the anchoring effect in emotion intensity judgment
Source: BMC Psychol. 2023 May 15;11:158. doi: 10.1186/s40359-023-01101-w (PMC10186746; doi:10.1186/s40359-023-01101-w)
Supplement: Supplementary file 2 — Supplementary Material 2 [file 40359_2023_1101_MOESM2_ESM.docx]

# Supplement

**The pilot study—the process of compiling material, rating and screening**

The pilot study was used to design the interpersonal emotion scenarios used for the emotion judgment tasks in the formal experiments. The pilot study included (1) interviewing younger and older adults about their daily positive and negative emotions and the corresponding emotional events; (2) compiling the interpersonal emotion scenarios based on the interview results; and (3) screening out the required material pools for formal experiments with respect to characteristics such as familiarity and the importance of the emotional event.

**The interview about daily emotional events**

Thirty-two younger adults (age: M=23.34; SD=1.28) and twenty-eight older adults (age: M=65.11; SD=3.91) were recruited for interviews. The participants were initially asked to report their daily frequently occurring emotional events and the emotion they felt in that event, and each person reported 3 positive events and 3 negative events. Joy and pride were selected as positive emotions (frequency ratio of the elderly: joy 76.25%, pride 20%; frequency ratio of the young: joy 63.27%, pride 21.43%), and anger, distress and sadness were selected as negative emotions in the formal experiment (frequency ratio of the elderly: anger 8.45%, distress 46.48%, sadness 8.45%; frequency ratio of the young: anger 22.22%, distress 37.37%, sadness 14.14%), according to the top frequency among the two groups. Although the frequency of guilt was 16.16% for younger adults, anger, distress and sadness accounted for relatively high proportions in both age groups and were selected to better compare the characteristics of the two age groups. The highly frequent emotional event themes mentioned were used for later scenario material compiling.

Participants were then required to rate the frequency with which their emotions were biased, perceived and judged by others in daily life (from 1 “never” to 5 “always”). The mean rating score of younger adults was M=2.84 (SD=0.81), and that of older adults was M=2.32 (SD=0.61). Finally, participants were asked to rate the degree of influence of this category of biased emotional judgment event on their life (from 1 “no influence” to 5 “strong influence”). The mean rating score of younger adults was M=2.91 (SD=0.86), and that of older adults was M=2.14 (SD=0.76). According to these results, the frequency and impact of such biased emotional judgment on the lives of younger and older adults are moderate. The frequency and influence of such events is significantly higher for young people than for older people, t_frequency_ (58)=2.97，p<.01; t_frequency_ (58)=3.64, p<.001. Thus, there may be age differences in the occurrence and subsequent impact of interpersonal bias in emotional judgments on the elderly and the young.

**Compiling the emotional scenario material**

After determining the emotion category for the experimental material, the emotional event theme and category were screened out according to the frequency at which they were mentioned in interviews. One or two emotional scenario themes for each emotion category that showed a high frequency in interviews were selected.

For older adults, the selected **joy-related scenario themes** were “interpersonal interaction” (frequency ratio: 32.50%, e.g., visits from children, meeting with friends) and “leisure activities” (frequency ratio: 17.50%, e.g., singing and dancing, playing chess). The selected **pride-related scenario themes** were “personal achievement” (frequency ratio: 11.25%, e.g., work achievement, winning a prize) and “achievement of children” (frequency ratio: 21.25%, e.g., children’s graduation, children’s job promotion). The selected **anger-related scenario themes** were “interpersonal relationship” (frequency ratio: 22.53%, e.g., spousal relationship, parent-child relationship) and “man-made accident” (frequency ratio: 23.94%, e.g., theft, fraud). The selected **distress-related scenario themes** were “interpersonal relationship” (frequency ratio: 22.53%, e.g., spousal relationship, parent-child relationship) and “accident caused by self” (frequency ratio: 23.94%, e.g., property loss). The selected **sadness-related scenario themes** were “interpersonal relationship” (frequency ratio: 22.53%, e.g., spousal relationship, parent-child relationship) and “health-related accident” (frequency ratio: 30.99%, e.g., disease in family, disease in self).

For younger adults, the selected **joy-related scenario themes** were “interpersonal interaction” (frequency ratio: 27.55%, e.g., help from friends, meeting with friends) and “leisure activities” (frequency ratio: 17.50%, e.g., traveling, watching concerts). The selected **pride-related scenario theme** was “personal achievement” (frequency ratio: 35.71%, e.g., obtaining a scholarship, fulfilling a hard task). The selected **anger-related scenario themes** were “interpersonal relationship” (frequency ratio: 27.27%, e.g., romantic relationship, friend relationship) and “man-made accident” (frequency ratio: 19.19%, e.g., property damage, fraud). The selected **distress-related scenario themes** were “study and work” (frequency ratio: 32.32%, e.g., spousal relationship, parent-child relationship) and “accident caused by self” (frequency ratio: 19.19%, e.g., stay up late to complete homework, working overtime). The selected **sadness-related scenario themes** were “study and work” (frequency ratio: 32.32%, e.g., examination failure, contest failure) and “interpersonal relationship” (frequency ratio: 30.99%, e.g., romantic relationship, parent-child relationship).

Then, we compiled the emotion scenarios required for the experiment for each scenario theme and category and for both younger and older adults. **All emotional events were compiled using the following standards:**

1. Scenario frame: **protagonist** + **details 1** (age, gender or other personal characteristics) + **details 2** (lifestyle habits and hobbies, have some correlation with the subsequent events) + **emotional event** (events able to elicit moderate levels of the target emotion)
2. Number of words: 30-50 words in Chinese

(3) Protagonists’ gender and age ensured a certain balance in the overall situation.

An example of a joy-related event scenario is as follows:

**Example scenario for older adults:** Ms. Li, a 70-year-old woman, has been living alone for many years. Her daughter will visit her during the Mid-Autumn Festival.

**Example scenario for younger adults:** Ming has just started college. None of his former friends live in the same city. Last week, one of his high school classmates visited him.

We compiled 100 emotional scenarios for both younger adults and older adults (20 scenarios for each emotion). These scenarios were rated by another group of recruited participants. **The calibration group**, including sixty-one older adults (age: M=65.08; SD=6.48; education year: M= 10.41, SD= 2.85; 23 males) and sixty-three younger adults (age: M=22.02; SD=3.28; education year: M= 14.87, SD= 2.64; 30 males), rated the scenarios in terms of **familiarity** (the extent to which you have encountered or seen such incidents in your daily life) and **importance** (the importance attached to the occurrence and handling of such incidents) on a seven-point Likert scale (from 1 = Not familiar/important at all to 7 = Extremely familiar/important) and then judged the emotion felt by the protagonist in the scenario for a given emotion category ( “yes” or “no”) and estimated the **emotion intensity** (0-100) felt by the protagonist in the scenario for given emotion category. The calibration group’s mean rating of emotion intensity would be further used in formal study to calculate the judgment accuracy.

**Emotional scenario material screening**

After collecting the rating data, the emotional scenarios were screened to ensure no significant difference between the older group and the younger group in the evaluation of importance, familiarity and emotional intensity. **The screening standard was as follows:**
(1) Screening based on participants: participants with significant optimistic/pessimistic bias were screened out (the average intensity of positive emotions differs from that of negative emotions by more than 20).

(2) Screening based on emotional scenario: emotional scenarios with five or more negative answers were screened out; emotional scenarios with an extremely high or low average intensity rating were screened out (intensity rating was above 90/below 50).

Finally, 66 emotional scenarios from the elderly and 70 emotional scenarios from the young were used as the final material set for the formal experiment. The descriptive information of the mean rating on the emotion intensity scale, familiarity and importance in each age group are shown in Table S1. The calibration group’s ratings of the scenario materials had nonsignificant differences in age on the rating of intensity, familiarity, and importance. Familiarity rating: F(1,122)=0.025, p=0.875, η_p_^2^=0; importance rating: F(1,122)=0.001, p=0.979, η_p_^2^ =0; intensity rating: F(1,122)=0.026, p=0.873, η_p_^2^ =0.

**Table S1**

*Description of familiarity, importance, and emotional intensity for young and older adults in positive and negative emotional scenarios*

| Measures | Emotional valence | Old | | Young | |
| --- | --- | --- | --- | --- | --- |
|  |  | M | SD | M | SD |
| Familiarity | Positive emotion | 4.85 | 1.05 | 4.68 | 0.94 |
| Familiarity | Negative emotion | 4.58 | 1.31 | 4.70 | 1.02 |
| Importance | Positive emotion | 4.98 | 0.78 | 4.89 | 0.77 |
| Importance | Negative emotion | 4.76 | 1.07 | 4.84 | 0.96 |
| Emotion intensity | Positive emotion | 67.88 | 11.07 | 67.78 | 11.33 |
| Emotion intensity | Negative emotion | 66.39 | 14.16 | 66.11 | 14.55 |
